# Assessing the effect of patient screening and isolation on curtailing *Clostridium difficile* infection in hospital settings

**DOI:** 10.1186/s12879-017-2494-6

**Published:** 2017-06-02

**Authors:** Sara Maghdoori, Seyed M. Moghadas

**Affiliations:** Agent-Based Modelling Laboratory, York University, Toronto, ON M3J 1P3 Canada

**Keywords:** *Clostridium difficile*, Colonization, Transmission dynamics, Screening, Reproduction number

## Abstract

**Background:**

Patient screening at the time of hospital admission is not recommended as a routine practice, but may be an important strategy for containment of *Clostridium difficile* infection (CDI) in hospital settings. We sought to investigate the effect of patient screening in the presence of asymptomatic carriers and in the context of imperfect patient isolation.

**Methods:**

We developed and parameterized a stochastic simulation model for the transmission dynamics of CDI in a hospital ward.

**Results:**

We found that the transmission of CDI in the hospital, either through asymptomatic carriers or as a results of ineffective implementation of infection control practices, at the time of hospital admission. The results show that, for a sufficiently high reproduction number of CDI, the disease can persist within a hospital setting in the presence of in-ward transmission, even when there are no asymptomatically colonized patients at the time of hospital admission.

**Conclusions:**

Our findings have significant public health and clinical implications, especially in light of the emergence and community spread of hypervirulent CDI strains with enhanced transmission rates and toxin production. Rapid detection of colonized patients remains an important component of CDI control, especially in the context of asymptomatic transmission. Screening of in-hospital patients with potential exposure to colonized patients or contaminated environment and equipment can help reduce the rates of silent transmission of CDI through asymptomatic carriers.

**Electronic supplementary material:**

The online version of this article (doi:10.1186/s12879-017-2494-6) contains supplementary material, which is available to authorized users.

## Background

*Clostridium difficile* infection (CDI) has become the leading cause of hospital acquired nosocomial diarrhea worldwide [1, 2], and remains among the top 10 infectious causes of death in the developed world [3–5], with alarming rates in terms of morbidity and mortality [6]. The severity of CDI ranges from asymptomatic and mild diarrhea to life-threatening conditions including toxic, megacolon, bowel perforation and sepsis [7]. The steady increase in the incidence of CDI along with prolonged hospital stays inflict a substantial impact on the healthcare systems in terms of costs and patient outcomes [8, 9]. The changing epidemiology of CDI [10], especially in the presence of hypervirulent strains [11], underscores the need for improved and strategically integrated infection control measures.

For many nosocomial infections including *C. difficile*, hospitals and other healthcare facilities are primary resources for disease control, but may also serve as ‘hot spots’ for disease transmission, with subsequent hospital-to-community spread. Therefore recommended measures and strategies for diagnosis and management of patients with CDI are mainly implemented in the healthcare facilities [12, 13]. In these settings, often several interventions are implemented simultaneously, making it difficult to estimate the importance and effectiveness of each intervention individually or relative to another [13]. A number of modelling studies on the transmission dynamics of *C. difficile* in hospital settings have evaluated the effects of various interventions, including screening of patients at the time of hospital admission and within-ward transmission reduction measures such as isolation [14–17]. While projecting the potential gains that can be achieved through these interventions, these models indicate different ranks for each intervention in terms of their importance and effectiveness on reducing the incidence of CDI [14–16]. A recent systematic review of mathematical models of CDI and colonization in healthcare settings shows a substantial variability in the natural history assumptions, outcome measures presented, and interventions examined in published studies, highlighting the challenge in identification of optimal intervention strategies to control and prevent CDI transmission [18].

In this study, we sought to develop a stochastic model of *C. difficile* transmission dynamics in a hospital setting, based on biological and epidemiological characteristics of this disease including its natural history [14, 15, 19], to investigate the effect of screening patients on reducing the prevalence and incidence of CDI. We included several key parameters into the model, representing the level of patient screening, effectiveness of isolation, treatment failure, and the level of susceptibility to infection. While the contribution of admitted patients who are colonized without presenting symptoms (referred to as asymptomatic carriers) to the spread of CDI has been recognized [14, 20], the effect of imperfect isolation (i.e., *<* 100% effective in preventing *C. difficile* transmission in the ward) and screening of in-hospital patients with exposure to CDI have not been accounted for. By inclusion of these factors in the model, we simulated various scenarios for detection and isolation of colonized patients, and ranked the model parameters in terms of their relative importance on the reduction of CDI incidence and prevalence.

## Methods

### Biological and epidemiological assumptions

Clinical studies demonstrate that a significant fraction of colonized patients remain infectious and can transmit the bacterium with no apparent symptoms [12, 14, 21]. These individuals may develop immune responses against *C. difficile* [14, 21], and we assumed that only colonized patients without immune responses develop clinical symptoms. It is well documented that the use of antibiotics increases susceptibility to colonization of *C. difficile,* as a result of damage to gut flora [14, 22, 23]. High rates of treatment failure for the management of CDI have been reported [14, 24, 25], with the possibility of recurrence even after effective treatment [26, 27]. We considered timelines for detecting colonized patients in two types of laboratory diagnosis methods including (i) the real-time polymerase chain reaction (PCR), and (ii) other tests of enzyme immunoassays, stool culture, and nucleic acid amplification [28–30]. The average time to reportable results for rapid PCR (about 1.5 h) is significantly shorter compared to other testing assays that generally range from 1 to 3 days [28, 31, 32].

### The model

**Fig. 1:**
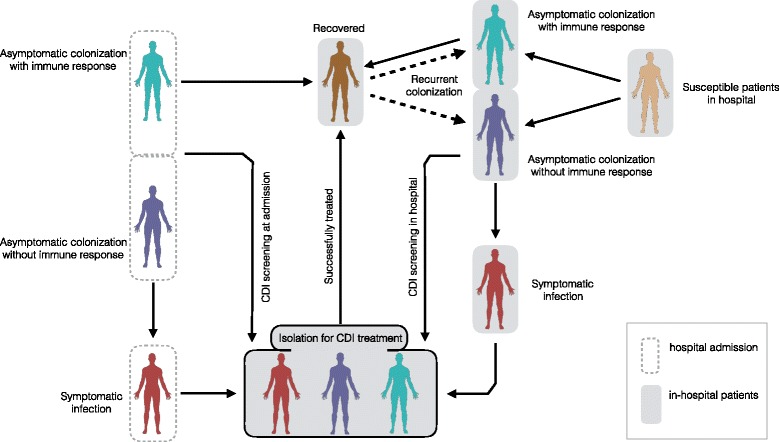
Model structure with transitions between compartments, corresponding to the epidemiological statuses of individuals at the time of hospital admission and in-hospital patients

In order to develop the general framework of the model, we divided the population in a single ward into several compartments, including the classes of individuals who are susceptible and colonized. We further divided susceptible individuals into two main categories: those who are currently receiving antibiotic treatment for the management of infections other than CDI, with possible damage to their gut flora and increased risk of *C. difficile* colonization (*S*_+_); and those without recent exposure to antibiotic treatment (*S*_−_). We considered a transition between the two susceptible classes based on the rates of antibiotic treatment and recovery from damaged gut flora. A fraction of colonized individuals will mount immune responses against *C. difficile* and move to the class *C* ^+^; the remaining will enter the class *C* ^−^ without developing immune responses, and will progress to symptomatic disease (*I*). Both *C* ^+^ and *C* ^−^ comprised of colonized patients who are asymptomatically infectious. Patients with clinical symptoms are diagnosed and isolated. We assumed that patients with *C. difficile* symptoms under treatment are not discharged from the hospital prior to the resolution of their symptoms. Successful treatment of symptomatic CDI will lead to the resolution of symptoms and full recovery. The schematic model diagram for the dynamics of infection and patient screening is shown in Fig. 1.

Recognizing the importance of asymptomatic carriers in the hospital transmission of CDI [14, 20], we extended the main structure to include screening of patients at the time of admission, and in-hospital patients. A fraction of patients at the time of hospital admission will be screened and isolated if diagnosed with colonization of *C. difficile*, including patients with apparent symptoms (Additional file 1: Figure S1). For diagnosis using PCR testing method, we omitted the short time-interval (approximately 1.5–2 h [31]) prior to the release of laboratory results. We therefore introduced two classes of patients who are isolated and treated upon screening, based on whether they develop immune responses (${C}_T^{+}$) or not (${C}_T^{-}$). For diagnosis using other testing methods, we accounted for the delay between the time of sample collection and the time when laboratory results are released. Considering such a time delay, we introduced two additional classes of patients under screening with immune responses (${D}_T^{+}$) and without immune responses (${D}_T^{-}$). We assumed that during the screening period before the laboratory results are released, patients are neither isolated nor treated for *C. difficile* [13]. The transition of these patients to ${C}_T^{+}$ and ${C}_T^{-}$ for treatment of *C. difficile* occurs after the release of positive laboratory testing. Screening of in-hospital patients is considered for those with potential exposure to CDI through colonized patients, staff, or contaminated environment and equipment. In this model, we assumed that the same laboratory testing method was used for screening of patients at the time of admission and in-hospital patients.

The effects of treatment, screening, and the development of immune responses upon colonization are included in the reduction of disease transmission and transitions between different model compartments. Considering a mass-action incidence for disease transmission with homogenous mixing, the model can be expressed by systems of differential equations (see Additional file 1).

### The basic reproduction number

The basic reproduction number, commonly denoted by *R*_0_, is defined as the average number of secondary cases generated by an infectious case introduced into an entirely susceptible population [33]. We applied the next generation method [33] to calculate the reproduction number in terms of the model parameters. Using this method in the absence of treatment or screening (see Additional file 1), we obtain the basic reproduction number in terms of the model parameters:

$$ {R}_0=\frac{\mathrm{f}\kappa \upsilon \beta r{b}_s N}{\mu +\gamma}\left(\frac{\psi \left(\mu +{\tau}^{+}-\alpha \mu \right)+\alpha \mu +{\tau}^{-}}{\mu \left(\mu +{\tau}^{+}+{\tau}^{-}\right)}\right)+\frac{\left(1- f\right)\kappa \beta r{b}_s N}{\mu +\epsilon}\left(\frac{\psi \left(\mu +{\tau}^{+}-\alpha \mu \right)+\alpha \mu +{\tau}^{-}}{\mu \left(\mu +{\tau}^{+}+{\tau}^{-}\right)}\right)+\frac{\left(1- f\right)\epsilon \beta r{b}_s N}{\mu \left(\mu +\epsilon \right)\left(\rho +{\mu}_I\right)} $$

The description of the parameters in the expression for *R*_0_ is provided in Table 1. In this expression, *β* represents the baseline transmission rate for patients with CDI symptoms. Given a reproduction number, the transmission rate in simulated scenarios was calculated from the above expression while fixing other parameters.

**Table 1:** Description of model parameters and their associated values (ranges)

Parameter	Description	Value (range)	Source
*R*_0_	basic reproduction number	1.07 (0.55–1.99) 2.6 (1–7)	[14] [34]
*r*	hospital admission rate	0.17 day^−1^	assumed to be the same as discharge rate
*δ*	fraction of admitted patients with CDI symptoms	1.2 × 10^−4^	Calculated based on the rate of 6.9 per 10,000 patient-days estimated over 82 periods of 4 weeks [20]
*θ*	fraction of admitted patients without CDI symptoms who are screened	0.925 (0–1)	[20]
*b*_*s*_	fraction of screened admitted patients who are susceptible	0.952 (0–1)	[20]
*η*	fraction of screened patients who are colonized and develop immune responses	0.6 (0.45–0.75)	[14, 15]
*α*	fraction of screened admitted patients who are susceptible and receive antibiotic treatment	0.22 (0.15–0.29)	[14]
*σ*	fraction of in-hospital patients who are screened following exposure to CDI	0.9 (0–1)	assumed, varied in sensitivity analysis
*f*	fraction of colonized patients who develop immune responses	0.6 (0.45–0.75)	[14, 15]
*ε*	rate of developing CDI symptoms in colonized patients	0.2 (0.14–0.26) day^−1^	[14, 15]
*τ* ^+^	recovery rate of damaged gut flora	0.011 day^−1^	[37]
*τ* ^−^	rate of antibiotic treatment damaging gut flora	0.11 day^−1^	[36]
*q*	fraction of CDI patients who are successfully treated	0.8 (0.56–1)	[14, 15]
*μ*	discharge rate of hospital patients without symptomatic infection	0.17 day^−1^	[14, 15]
*μ*_*I*_	CDI-caused death rate	0.0012 (0.001–0.01) day^−1^	[14, 15]
*ρ*	rate of symptoms resolution for CDI patients under treatment	0.25 (0.143–0.33) day^−1^	[35]
*γ*	recovery rate of CDI patients under treatment after symptoms resolution	0.2 (0.143–0.33) day^−1^	[14, 15]
*κ*	relative transmissibility of colonized patients without symptoms	0.5 (0.3–0.7)	assumed, varied in sensitivity analysis
*ν*	reduction of transmissibility due to immune responses	0.5 (0.3–0.7)	assumed, varied in sensitivity analysis
*ξ*	effectiveness of isolation for CDI patients	0.8, 0.9, 1 (0.8–1)	assumed, varied in sensitivity analysis
Ψ	reduced risk of CDI in patients without antibiotic exposure	0.2 (0.06–0.55)	[24]
*π*	time-interval between sample collection and release of laboratory results	1 (1–3) days	[28, 31, 32]

### Stochastic model implementation

We implemented the model stochastically, and used the Gillespie direct algorithm to simulate the stochastic model within a (non-ICU) hospital ward with 50 beds (as the population size) within the range of previous studies for *C. difficile* [18]. To estimate the transition time between the two consecutive events in the stochastic process, we let *dt* =  − log(*n*_1_)/*Δ*, where *n*_1_ is a random number drawn from the uniform distribution on the unit interval [0*,* 1], and *Δ* is equal to the sum of the rates for all possible events. We then ordered the events as an increasing fraction of *Δ* and generated another uniform number *n*_2_ between 0 and 1 to determine the nature of the next event. We ran 500 independent simulations to calculate the average of sample realizations of the stochastic process in each scenario.

### Parameterization

To simulate the stochastic model, we extracted a number of parameter values from the previous literature. The mean values of these parameters were used for the main results, and the estimated ranges were explored in the sensitivity analyses to investigate the relative influence of each parameter on CDI prevalence. We varied *R*_0_ in the estimated ranges reported in two previous studies [14, 34]. Lanzas et al. [14] fitted a mathematical model to hospital data for patients between January and December 2008, during which *C. difficile* diagnosis was conducted by stool toxin, and estimated the mean reproduction number of 1.07 (range: 0.55–1.99). By linking the secondary cases to index cases using PCR ribotyping, Norén et al. [34] estimated the mean reproduction number of 2.6 (range: 1–7) for a single hospital ward data between February 1999 and January 2000. The transmission parameter was calculated based on a given *R*_0_, while fixing other parameters of the model. The rate of disease transmission by asymptomatic carriers or colonized patients who mount immune responses may be lower than those with CDI symptoms. While quantification for this lower transmission rate is lacking, we assumed a relative transmissibility that is reduced by 50% on average and considered a range of 30%–70% in the sensitivity analyses. The fraction of colonized patients who mount immune responses was set to 0.6 within the estimated range 0.45–0.75 [14, 15]. We considered a mean period of 5 days for colonized patients to develop symptoms [14, 15]. The duration of CDI symptoms was assumed to have a mean of 4 days [35], with an average of 5 days for full recovery after the resolution of symptoms [14, 15]. Treatment is assumed to be successful in 80% of CDI patients [14, 15]. Based on reported cases [20], we assumed 4.8% of the screened patients admitted to the hospital were colonized [28], and therefore 95.2% were assumed to be susceptible. We used the rate of 6.9 per 10,000 patient-days estimated over 82 periods of 4 weeks each to calculate the daily fraction 1.22 × 10^−4^ of patients admitted to the hospital presenting CDI symptoms [20]. We assumed 22% of screened patients who are susceptible to colonization receive antibiotic treatment [14]. The rate of in-hospital antibiotic treatment damaging gut flora was set to 0.11 per day [36], and the rate of recovery was assumed to be 0.011 per day [37]. The risk of antibiotic-associated *C. difficile* colonization relative to no antibiotic exposure has been reported to vary in a wide range of 1.8–16.8 [24, 38]. We considered a 5-fold higher risk of *C. difficile* colonization for patients under antibiotic treatment. The discharge rate (including mortality) is assumed to be 0.17 per day for hospital patients without CDI symptoms, with a CDI-induced mortality rate of 0.0012 per day for patients with *C. difficile* symptoms [14, 15]. The discharge rate corresponds to an average of 5.9 days for the length of hospital stay. For scenario evaluation, the fraction of patients screened at the time of admission was set to 0.925 [20]. This fraction for screening in-hospital patients with potential exposure to CDI was assumed to be 0.9. The effectiveness of patient isolation, represented by the parameter *ξ* in the model, was varied in the range 0.8–1. To account for the effect of imperfect isolation, we multiplied the factor (1 − *ξ*) by the baseline transmission (*β*) in the model equations (See Additional file 1). Parameter values and their respective ranges are provided in Table 1.

## Results

We ran the stochastic simulations for 200 days, with the introduction of two initial asymptomatic infections *C* ^+^(0) = 1 and *C* ^−^(0) = 1. We considered two different reproduction numbers (*R*_0_ = 1.07, 2.6) in the estimated ranges of previous studies [14, 34]. The results for *R*_0_ = 1.07 are described here. The corresponding simulation results for *R*_0_ = 2.6 are summarized in the Additional file 1.

### Model with rapid laboratory testing

**Fig. 2:**
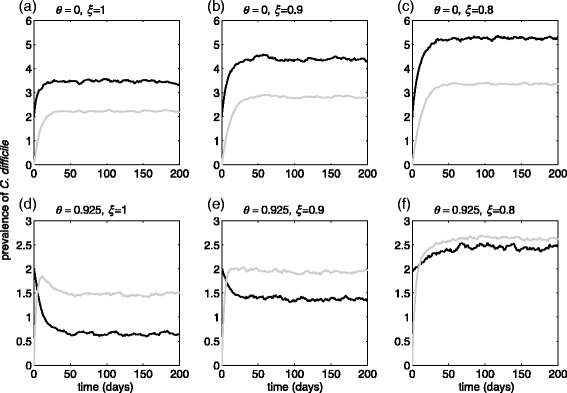
Prevalence of *C. difficile* with *R*_0_ = 1.07 over 200 days in the model with rapid testing, without screening (**a**-**c**) and with screening (**d**-**f**) 92.5% of patients at the time of hospital admission. Curves represent the prevalence of undiagnosed colonized patients (*black*), and isolated patients (*grey*) under CDI treatment. Total prevalence is the sum of *black* and *grey curves*. The effectiveness of CDI patient isolation in preventing in-ward transmission was 100% (**a**, **d**), 90% (**b**, **e**), and 80% (**c**, **f**)

For the mean value of *R*_0_ = 1.07 in the range 0.55–1.99 [14], Fig. 2 shows the prevalence of *C. difficile* for three scenarios in which the effectiveness of patient isolation in preventing infection transmission is 100% (Fig. 2a, d), 90% (Fig. 2b, e), and 80% (Fig. 2c, f). In these simulations, the baseline scenario without screening (*θ* = 0) was compared with the scenario of 92.5% screening at the time of hospital admission. When the effectiveness of patient isolation is 100%, the prevalence of *C. difficile* (i.e., the sum of black and grey curves) is reduced from 5.7 cases without screening to 2.1 cases (on average) with 92.5% screening of patients at the time of admission. This corresponds to 63.2% reduction of prevalence 50 days after the start of screening. For imperfect isolation with less than 100% effectiveness, the benefits of screening and detection of colonized patients are reduced as a result of within-ward transmission. For example, an isolation measure with 90% effectiveness reduces the prevalence by 54.2% from an average of 7.2 cases without screening to 3.3 cases with screening. When the effectiveness of patient isolation is further reduced to 80%, the percentage reduction of the prevalence of *C. difficile* with screening is at 41%. Results for a higher reproduction number (*R*_0_ = 2.6) show that the reduction of the prevalence is substantially reduced for the corresponding scenarios (Additional file 1: Figure S3).

### Model with time-delay in laboratory testing

**Fig. 3:**
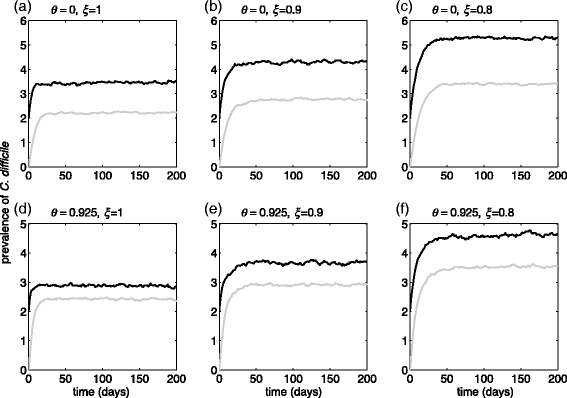
Prevalence of *C. difficile* with *R*_0_ = 1.07 over 200 days in the model with time delay between sample collection and the release of laboratory results, without screening (**a**-**c**) and with screening (**d**-**f**) 92.5% of patients at the time of hospital admission. Curves represent the prevalence of undiagnosed colonized patients (*black*), and isolated patients (*grey*) under CDI treatment. Total prevalence is the sum of *black* and *grey curves*. The effectiveness of CDI patient isolation in preventing in-ward transmission was 100% (**a**, **d**), 90% (**b**, **e**), and 80% (**c**, **f**)

Figure 3 shows the prevalence of *C. difficile* when an average of 2 days is considered for time delay between sample collection and the release of laboratory results. Compared to the results for rapid testing, we observed significantly lower effect of screening on reducing the prevalence of CDI. When the effectiveness of patient isolation is 100%, the prevalence of *C. difficile* reduces from 5.7 cases (on average) without screening to 5.3 cases (on average) with 92.5% screening of patients at the time of hospital admission. This corresponds to only 7% reduction of prevalence 50 days after the start of screening. For an isolation strategy with 90% or 80% effectiveness, the percentage reduction of the prevalence of *C. difficile* with screening remains below 6%.

### Disease persistence without admission of colonized patients

**Fig. 4:**
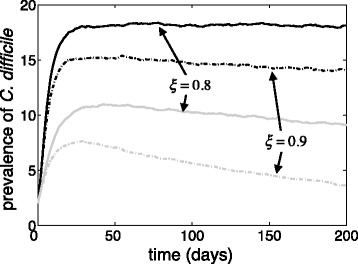
Prevalence of *C. difficile* with *R*_0_ = 1.8 (*grey curves*) and *R*_0_ = 2.6 (*black curves*) over 200 days with no admission of colonized patients (*b*_*s*_ = 1). The effectiveness of patient isolation in preventing hospital transmission was 80% (*ξ* = 0.8; *solid* curves) and 90% (*ξ* = 0.9; *dashed* curves)

Our simulations show that, for a sufficiently high transmissibility of CDI, the disease persists within a hospital setting in the presence of in-ward transmission, even when there are no asymptomatically colonized patients at the time of hospital admission. Figure 4 shows the persistence of CDI for *R*_0_ = 1.8 (grey curves) and *R*_0_ = 2.6, (black curves) when hospital admission occurs only through susceptible compartments in the model (i.e., *b*_*s*_ = 1). This suggests that the admission of asymptomatically colonized patients is not the sole factor in persistence of CDI in hospital settings.

### Relative reduction of CDI incidence

**Fig. 5:**
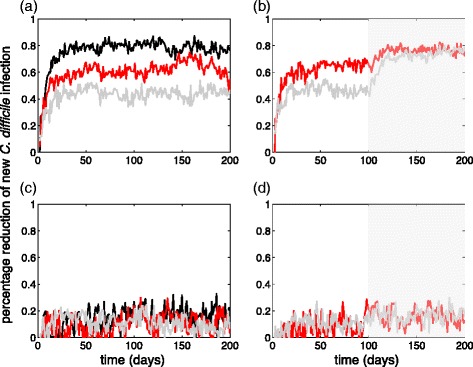
Percentage reduction in the number of new *C. difficile* infection with *R*_0_ = 1.07 over 200 days in the model with rapid testing (**a**, **b**) and the model with time delay between sample collection and the release of laboratory results (**c**, **d**). Curves in panels (**a**) and (**c**) represent the reduction achieved with screening 92.5% of patients at the time of hospital admission, where the effectiveness of patient isolation in preventing infection transmission in hospital is: 100% (*black*); 90% (*red*); and 80% (*grey*). Curves in panels (**b**) and (**d**) represent the reduction achieved with screening 92.5% of patients at the time of hospital admission, where the effectiveness of patient isolation in preventing infection transmission is: 90% (*red*); and 80% (*grey*). Screening 90% of in-hospital patients with exposure to CDI started on day 100 (*shaded* area)

We also evaluated the percentage reduction achieved in the number of new infections (i.e., the incidence) over the period of 200 days. Figure 5a (black curve) shows that when the effectiveness of patient isolation is 100% in preventing infection transmission in the model with rapid testing, the daily incidence of *C. difficile* is reduced by over 79% [95% CI: 78% – 79.6%] as a result of 92.5% screening at the time of hospital admission. With lower effectiveness of isolation, this reduction is decreased (Fig. 5a, red and grey curves) to 62.6% [95% CI: 61.8% – 63.4%] (and 44.2% [95% CI: 43.5% – 44.9%]) for patient isolation with effectiveness of 90% (and 80%).

For the model with an average time-delay of 2 days between sample collection and the release of laboratory results, the relative reduction of incidence is significantly lower at 16.3% [95% CI: 15.1% – 17.4%], 10.2% [95% CI: 9.0% – 11.5%], and 10.1% [95% CI: 9.4% – 11.2%] when the effectiveness of patient isolation is 100%, 90%, and 80%, respectively (Fig. 5c).

### Inpatients screening

For the scenarios in which patient isolation was less than 100% effective in preventing in-ward transmission, we implemented screening of inpatients with exposure to *C. difficile* in addition to screening of patients at the time of admission (Fig. 5b, d). For the model with rapid diagnostic testing, Fig. 5b shows the percentage reduction of the incidence of *C. difficile*, when screening 90% of in-hospital patients started on day 100. This resulted in an increasing trend in the percentage reduction of *C. difficile* incidence over time, reaching levels over 76% and comparable to those achieved with screening patients only at the time of hospital admission when the effectiveness of patient isolation is assumed to be 100% (Fig. 5a, black curve). However, simulating the model with a 2-day delay in release of laboratory results indicates little additional benefits, achieving only 6% increase in the relative reduction of CDI incidence by inpatient screening (Fig. 5d). When the effectiveness of patient isolation is lower at 80%, the effect of inpatient isolation becomes more pronounced (Fig. 5b, grey curve) in the model with rapid testing.

Similar results were obtained for the prevalence of CDI. Additional file 1: Figure S2 shows that, in the model with rapid testing, the prevalence of CDI reduces and stabilizes at a lower level compared to the level achieved prior to 100 days with screening patients only at the time of hospital admission (Additional file 1: Figure S2A,B). However, for the model with time delay in laboratory testing, we observed virtually no change in the prevalence of CDI after implementing inpatients screening (Additional file 1: Figure S2C,D).

### Sensitivity analysis

Our analysis (detailed in Additional file 1) reveals that for a low reproduction number (*R*_0_ = 1.07), the fraction of admitted patients who are susceptible to colonization (*b*_*s*_; PRCC > 0.80, *p*-value < 0.001) and the fraction of patients who are screened at the time of hospital admission (*θ*; PRCC > 0.7, *p*-value < 0.001) have the highest impact on the CDI prevalence based on their PRCC values; both of which are negatively correlated with the response (Additional file 1: Table S1). However, for a higher reproduction number (*R*_0_ = 2.6), the risk of acquiring infection as a result of exposure to antibiotics (*ψ*; PRCC > 0.85, *p*-value < 0.001) was the parameter with the largest impact (Additional file 1: Table S2). The effect of immune responses on reducing disease transmission (*υ*; PRCC < − 0.87, *p*-value < 0.001) has a strong effect and negatively correlated with the response in all scenarios. With rapid laboratory testing, parameters with moderate effects on the response include the effectiveness of patient isolation and the probability of successful CDI treatment. When laboratory testing was associated with a time-delay, additional parameters with moderate effects include the relative transmissibility of asymptomatic carriers and the relative risk of colonization with antibiotic exposure. Furthermore, the time-interval between sample collection and the release of laboratory results had a low to moderate effect on the response. The relative influence of model parameters with *R*_0_ = 1.07 is summarized in Table 2. These results suggest that several factors contribute to the ‘silent transmission’ of *C. difficile* through asymptomatic carriers, which is the main driver of infection spread in the hospital [39].

**Table 2:** Relative influence of the model parameters on the response (i.e., CDI prevalence) based on their PRCC indices and *p*-values below the significance level in the sensitivity analyses with *R*_0_ = 1.07

Model	relative influence					
	rapid laboratory testing			time-delay in laboratory testing		
Parameter	Strong	Moderate	Weak	Strong	Moderate	Weak
*f*			•			•
*κ*			•		•	
*ν*	•			•		
Ψ			•		•	
*σ*			•			•
*θ*	•					•
*q*		•			•	
*ρ*			•			•
*γ*			•			•
*ξ*		•			•	
*b*_*s*_	•			•		
*η*			•			•
*π*					•	

## Discussion

Our results show that the transmission of *C. difficile* within the hospital remains a key epidemiological parameter that can significantly influence the disease dynamics. We evaluated various scenarios in the presence of screening of patients at the time of hospital admission and in-hospital patients with potential exposure to *C. difficile* that may be due to person-to-person contacts or contaminated environment and equipment. For a relatively low reproduction number, we observed that when the effectiveness of CDI interventions was less than 100%, screening of in-hospital patients can lead to a reduction of *C. difficile* incidence over time, which may be comparable to that achieved with screening of patients only at the time of hospital admission when isolation is 100% effective in blunting disease transmission in the ward. However, the additional benefits of inpatient screening become negligible for a sufficiently high reproduction number. Furthermore, the silent transmission of *C. difficile* from asymptomatic carriers is an important pathway for the spread of CDI, especially among individuals with an increased risk of susceptibility to colonization.

Despite lower transmission rate for asymptomatic carriers, our results indicate that asymptomatic transmission in the hospital should be accounted for when designing and evaluating control interventions. Recent studies have evaluated the effectiveness of various intervention measures in healthcare facilities, including infection control practices [3, 12, 18, 40, 41]. A large population-based retrospective cohort study of all patients admitted to acute care hospitals between April 2011 and March 2012 in Ontario, Canada, found that selected hospital prevention strategies had limited effectiveness or were ineffectively implemented [3]. A controlled quasi-experimental study in the province of Quebec, Canada, evaluated the effect of identification and isolation of asymptomatic carriers on the incidence of healthcare associated CDI over a 15-month period. The findings showed that screening of admitted patients and isolating asymptomatic carriers decreased the incidence of CDI by 62.4% [20]. Collectively, these studies suggest that the effective implementation of measures to block the silent transmission of CDI by asymptomatically colonized patients remain essential components of containment strategies within healthcare facilities.

Previous studies have concluded, using modelling and simulation scenarios, that the admission of colonized patients and asymptomatic carriers are the main impediments to the control of CDI in healthcare facilities [14–17]. Our results in evaluating the effect of interventions concur with this conclusion. However, contrary to previous findings [14], we have shown that transmission within the ward alone can sustain new *C. difficile* colonizations in the range estimated for the reproduction numbers, even in the absence of colonized patients at the time of hospital admission. Our findings indicate that if infection control measures are implemented inefficiently, within-ward transmission can potentially offset the benefits of patient screening. For patient screening, we implemented the model taking into account the possible time-interval for detecting *C. difficile* using various laboratory testing methods. When colonized patients are not isolated during the time-interval prior to the release of laboratory tests, we observed that the effect of screening on the incidence of CDI is substantially reduced, even when patient isolation is 100% effective in preventing in-ward transmission. In an exploratory analysis, we found that if screening of patients at the time of hospital admission and screening of in-hospital patients are implemented individually, then the former would always outperform the latter in terms of reducing the prevalence and incidence of CDI irrespective of the reproduction number, time-delay in the release of laboratory tests, or effectiveness of patient isolation. These findings harken back to the importance of transmission by asymptomatically colonized patients.

Previous work also shows that improving environmental cleaning and hand hygiene leads to a substantial reduction of colonization rates [15]. In our study, while we have not explicitly modelled the effect of these measures, we evaluated how their effectiveness (represented in the *C. difficile* transmission parameter) influences the incidence and prevalence of CDI. This evaluation was performed using parameter estimates extracted from the published literature, which is subject to parameter uncertainty. Although the relative transmissibility of *C. difficile* for asymptomatic carriers who mount immune responses (compared to symptomatic CDI patients) has not been quantified, our sensitivity analyses support the robustness of the model outcomes with variation in parameter space. However, our model has several limitations including the measures for sensitivity and specificity of *C. difficile* laboratory tests. While we did not consider the variability in these measures documented in the literature [29, 32, 42], other parameters of the model could be adjusted to account for their effects. For example, the parameter representing the fraction of patients who are screened can be tuned to account for the level of sensitivity of a test for *C. difficile* detection. We assumed the same risk of acquiring *C. difficile* for all patients as a result of exposure to antibiotics; yet, we understand that the relative risk varies across different classes of antibiotics. This variation was explored through sensitivity analysis. Furthermore, we did not consider the variability in CDI transmission from environmental factors compared with person-to-person, which is affected by contact patterns of individuals. A recent data-driven study of individual movements in a long-term care facility demonstrates that the network of contacts in healthcare facilities is highly structured and deviates from random mixing [43]; an assumption that underlies many models in the literature including the one presented here. The structure of contacts among patients and staff in these settings could significantly affect the identification of optimal intervention strategies and their outcomes for the control of nosocomial infections [43]. The modelling framework developed here could be translated into an agent-based computational model to include individual characteristics and environmental factors, and assess the likely outcomes of novel interventions.

## Conclusions

Our findings indicate that the effective implementation of bundle strategies within hospital settings is critically important in the control of CDI even in the absence of asymptomatically colonized patients at the time of hospital admission. Furthermore, rapid detection of colonized patients can significantly affect the prevalence of CDI and its control, especially in the context of asymptomatic carriers and in-ward transmission. Further studies are required to quantify the effectiveness of current CDI interventions, and recurrent rates in hospital settings to parameterize decision models for the evaluation of preventive measures.

## Additional files

Additional file 1: Electronic Supplementary Information. (PDF 5994 kb)

Additional file 2: Matlab code for the model with no delay. (ZIP 2 kb)

Additional file 3: Matlab code for the model with delay. (ZIP 4 kb)

## Abbreviations

CDI: *Clostridium difficile* infection
PCR: Polymerase chain reaction
