# Supplementary material for: Assessing the effect of patient screening and isolation on curtailing Clostridium difficile infection in hospital settings
Source: BMC Infect Dis. 2017 Jun 2;17:384. doi: 10.1186/s12879-017-2494-6 (PMC5455129; doi:10.1186/s12879-017-2494-6)
Supplement: Supplementary file 1 — Electronic Supplementary Information. (PDF 5994 kb) [file 12879_2017_2494_MOESM1_ESM.pdf]

Supplemental Information
Assessing the effect of patient screening and isolation on curtailing *Clostridium difficile* infection in hospital settings

Sara Maghdoori, Seyed M. Moghadas
Agent-Based Modelling Laboratory, York University, Toronto, ON, Canada M3J 1P3

This Supplementary information provides details of model equations and additional simulations supporting the results discussed in the main text.

The model includes several compartments to represent the patient population in a hospital ward, including susceptible individuals under antibiotic treatment (S_+) and without exposure to antibiotic treatment (S_-); colonized patients who mount immune responses (C^+), colonized patients without immune responses (C^-); patients with symptoms of *C. Difficile* infection (I); diagnosed colonized patients from screening with immune response (C_T^+) and without immune responses (C_T^-). The schematic model diagram for the transitions between these classes of individuals are shown in Figure 1 of the main text. For the rapid laboratory testing, the model can be expressed as the following system of differential equations.

$$
\begin{aligned}
\frac{dS_+}{dt} &= (1-\delta)\alpha b_s rN - S_+(\Lambda + \Lambda_T) + \tau^- S_- - \tau^+ S_+ - \mu S_+ \\
\frac{dS_-}{dt} &= (1-\delta)(1-\alpha) b_s rN - \psi S_-(\Lambda + \Lambda_T) + \gamma\big(C^+ + qC_T^- + C_T^+\big) \\
&\quad + \tau^+ S_+ - \tau^- S_- - \mu S_- \\
\frac{dC^+}{dt} &= (1-\delta)(1-\theta)(1-b_s)\eta rN + f\big(S_+ + \psi S_-\big)\big(\Lambda + (1-\sigma)\Lambda_T\big) \\
&\quad - \gamma C^+ - \mu C^+ \\
\frac{dC^-}{dt} &= (1-\delta)(1-\theta)(1-b_s)(1-\eta) rN + (1-f)\big(S_+ + \psi S_-\big)\big(\Lambda + (1-\sigma)\Lambda_T\big)
\end{aligned}
$$

$$
\begin{aligned}
&+(1-q)\gamma C_T^- - \epsilon C^- - \mu C^- \\
\frac{dC_T^+}{dt} &= (1-\delta)(1-b_s)\eta\theta r N + \sigma f[S_+ + \psi S_-]\Lambda_T \\
&- \gamma C_T^+ - \mu C_T^+ \\
\frac{dC_T^-}{dt} &= (1-\delta)(1-b_s)(1-\eta)\theta r N + \sigma(1-f)[S_+ + \psi S_-]\Lambda_T \\
&+ \rho I - \gamma C_T^- - \mu C_T^- \\
\frac{dI}{dt} &= \delta r N + \epsilon C^- - \rho I - \mu_I I
\end{aligned}
$$

where

$$
\begin{aligned}
\Lambda &= \beta(\kappa\nu C^+ + \kappa C^-) \\
\Lambda_T &= \beta(1-\xi)(\kappa\nu C_T^+ + \kappa C_T^- + I) \\
N &= S_+ + S_- + C^+ + C^- + C_T^+ + C_T^- + I
\end{aligned}
$$

When considering laboratory testing with time-delay, we included two compartments of D^+ and D^- to account for time-interval between sample collection and the release of laboratory results for colonized patients with and without immune responses, respectively. During this time-interval, patients are neither isolated nor treated for CDI, and therefore the possibility of in-ward transmission exists. In this case, the model can be expressed as

$$
\begin{aligned}
\frac{dS_+}{dt} &= (1-\delta)\alpha b_s r N - S_+(\Lambda + \Lambda_T) + \tau^- S_- - \tau^+ S_+ - \mu S_+ \\
\frac{dS_-}{dt} &= (1-\delta)(1-\alpha) b_s r N - \psi S_-(\Lambda + \Lambda_T) + \gamma\big(C^+ + qC_T^- + C_T^+\big) \\
&+ \tau^+ S_+ - \tau^- S_- - \mu S_- \\
\frac{dC^+}{dt} &= (1-\delta)(1-\theta)(1-b_s)\eta r N + f\big(S_+ + \psi S_-\big)\big(\Lambda + (1-\sigma)\Lambda_T\big) \\
&- \gamma C^+ - \mu C^+ \\
\frac{dC^-}{dt} &= (1-\delta)(1-\theta)(1-b_s)(1-\eta) r N + (1-f)\big(S_+ + \psi S_-\big)\big(\Lambda + (1-\sigma)\Lambda_T\big) \\
&+ (1-q)\gamma C_T^- - \epsilon C^- - \mu C^- \\
\frac{dD^+}{dt} &= (1-\delta)(1-b_s)\eta\theta r N + \sigma f[S_+ + \psi S_-]\Lambda_T - \pi D^+ \\
\frac{dD^-}{dt} &= (1-\delta)(1-b_s)(1-\eta)\theta r N + \sigma(1-f)[S_+ + \psi S_-]\Lambda_T - \pi D^- \\
\frac{dC_T^+}{dt} &= \pi D^+ - \gamma C_T^+ - \mu C_T^+
\end{aligned}
$$

$$\frac{dC_T^-}{dt} = \pi D^- + \rho I - \gamma C_T^- - \mu C_T^-$$
$$\frac{dI}{dt} = \delta r N + \epsilon C^- - \rho I - \mu_I I$$

where

$$\Lambda = \beta\left[\kappa\nu(D^+ + C^+) + \kappa(D^- + C^-)\right]$$
$$\Lambda_T = \beta(1-\xi)(\kappa\nu C_T^+ + \kappa C_T^- + I)$$
$$N = S_+ + S_- + C^+ + C^- + D^+ + D^- + C_T^+ + C_T^- + I$$

Screening is implemented at the time of hospital admission (Figure S1) or for in-hospital patients with exposure to *C. difficile*. Parameters of the model are described in Table 1 of the main text.

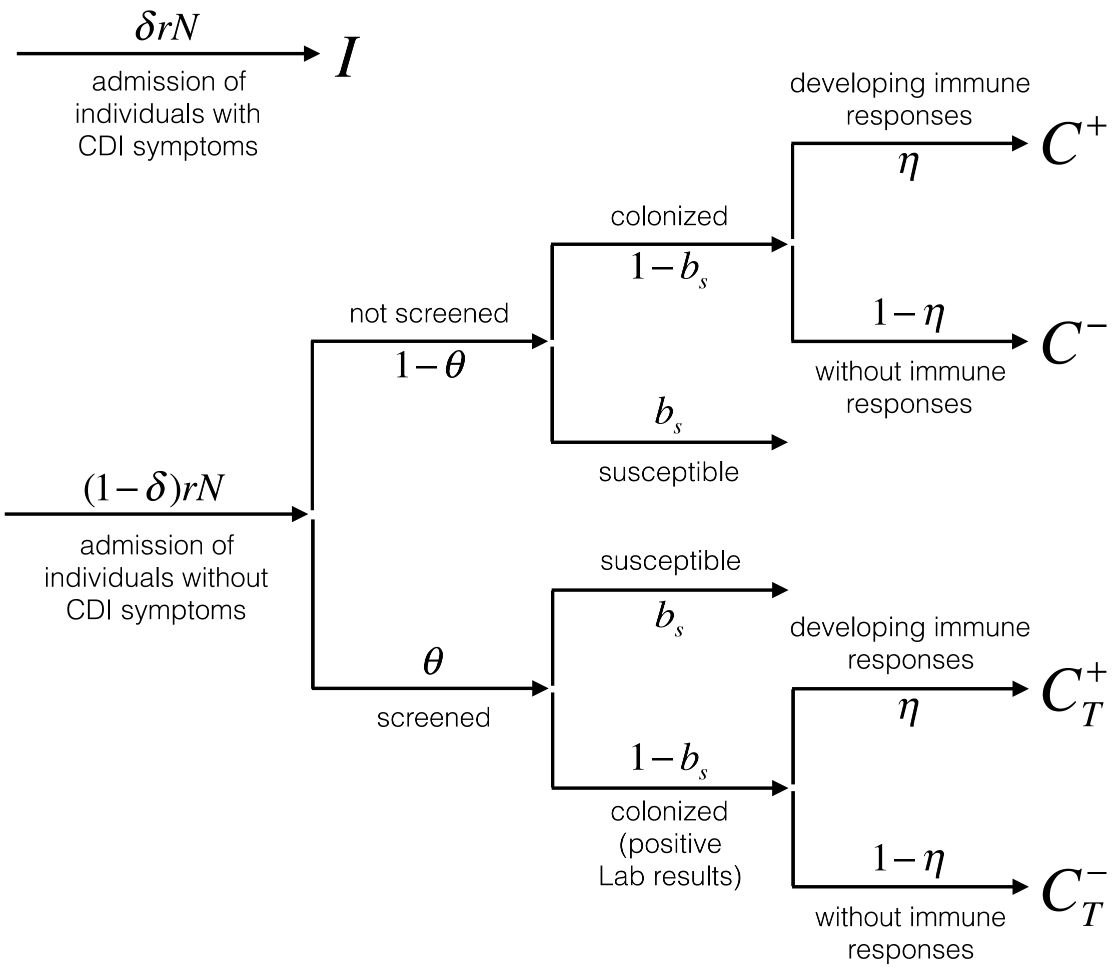

Figure S1: Schematic diagram for screening of patients at the time hospital admission.

Basic reproduction number

To calculate the basic reproduction number, we applied the next generation method (van den Driessche and Watmough, 2002), by representing F and V as the matrices for new infections and transitions in the infection subclasses

$$F = \begin{pmatrix} f(\Lambda+\beta I)S_+ + \psi f(\Lambda+\beta I)S_- \\ (1-f)(\Lambda+\beta I)S_+ + \psi(1-f)(\Lambda+\beta I)S_- \\ 0 \end{pmatrix} \tag{1}$$

and

$$V = \begin{pmatrix} -(1-b_s)\eta r N + \gamma C^+ + \mu C^+ \\ -(1-b_s)(1-\eta) r N + \epsilon C^- + \mu C^- \\ -\epsilon C^- + \rho I + \mu_I I \end{pmatrix} \tag{2}$$

Taking the Jacobian of F and V at the infection-free equilibrium, we obtain

$$J_F = \begin{pmatrix} f\beta\kappa\nu(\psi S_-^* + S_+^*) & f\beta\kappa(\psi S_-^* + S_+^*) & \frac{f\beta r b_s N}{\mu} \\ (1-f)\beta\kappa\nu(\psi S_-^* + S_+^*) & (1-f)\beta\kappa(\psi S_-^* + S_+^*) & \frac{(1-f)\beta r b_s N}{\mu} \\ 0 & 0 & 0 \end{pmatrix} \tag{3}$$

and

$$J_V = \begin{pmatrix} \gamma+\mu & 0 & 0 \\ 0 & \mu+\epsilon & 0 \\ 0 & -\epsilon & \rho+\mu_I \end{pmatrix} \tag{4}$$

where, at the infection-free equilibrium,

$$\begin{aligned} S_+^* &= \frac{r b_s N(\alpha\mu + \tau^-)}{\mu(\mu+\tau^+ + \tau^-)} \\ S_-^* &= \frac{r b_s N[(1-\alpha)\mu + \tau^+]}{\mu(\mu+\tau^+ + \tau^-)} \end{aligned} \tag{5}$$

According to the next generation method, the reproduction number is given by the spectral radius (dominant eigenvalue) of $J_F J_V^{-1}$. This gives

$$\begin{aligned} R_0 = {} & \frac{f\kappa\nu\beta r b_s N}{\gamma+\mu}\left(\frac{\psi(-\alpha\mu+\tau^+ + \mu) + \alpha\mu + \tau^-}{\mu(\mu+\tau^+ + \tau^-)}\right) \\ & + \frac{(1-f)\kappa\beta r b_s N}{\mu+\epsilon}\left(\frac{\psi(-\alpha\mu+\tau^+ + \mu) + \alpha\mu + \tau^-}{\mu(\mu+\tau^+ + \tau^-)}\right) \\ & + \frac{(1-f)\epsilon\beta r b_s N}{\mu(\mu+\epsilon)(\rho+\mu_I)} \end{aligned} \tag{6}$$

We used this expression to calculate the transmission rate of *C. difficile* for a given R_0, fixing all other parameter values. We note that in the absence of interventions, the model with time-delay reduces to the model without time-delay, and therefore has the same R_0.

Prevalence of CDI for $R_0 = 1.07$

Figure S2 shows the prevalence of *C. difficile* for the scenarios described in the main text.

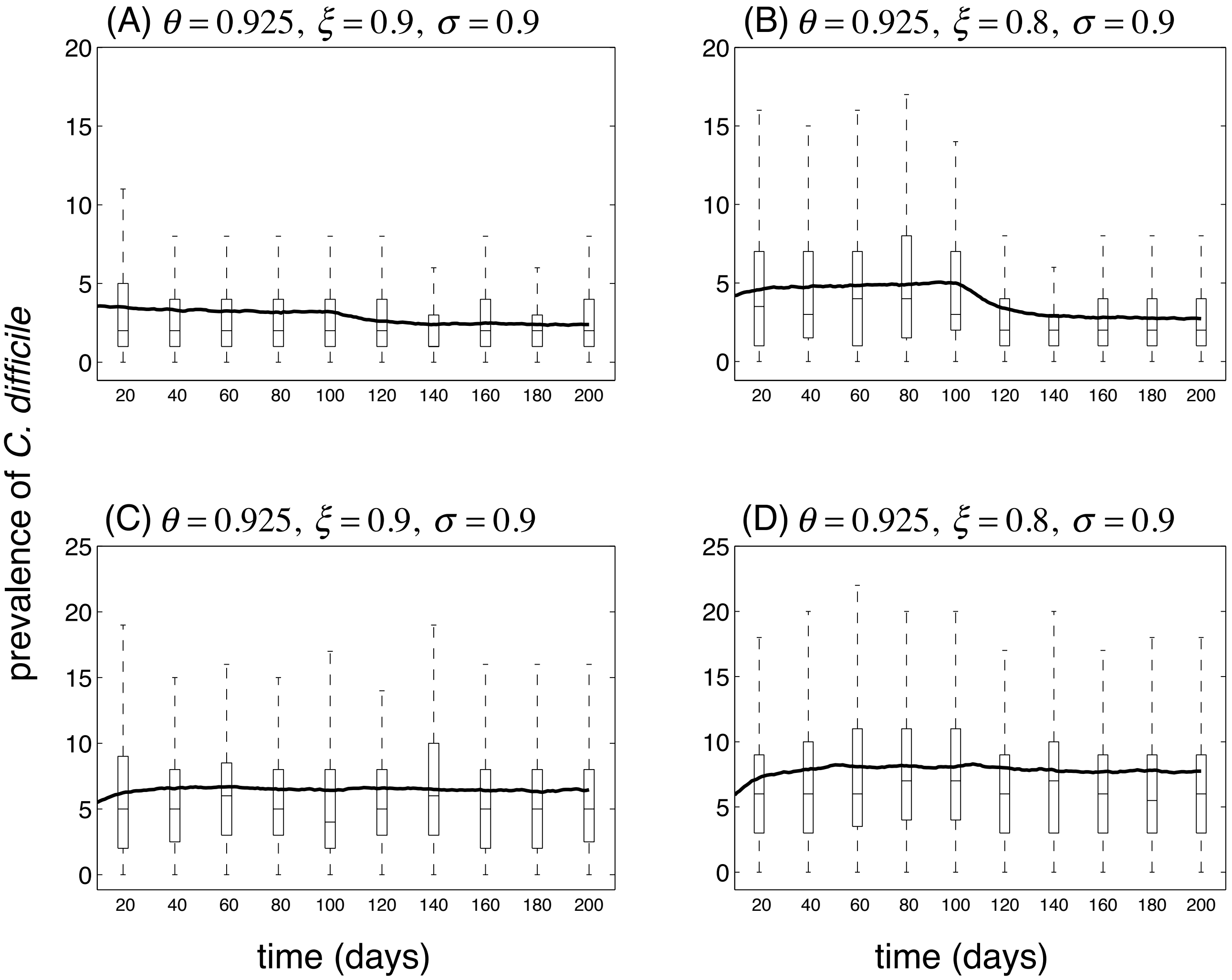

Figure S2: Prevalence of *C. difficile* with $R_0 = 1.07$ over 200 days for the model with rapid laboratory testing (A,B) and the model with a time-delay in laboratory testing (C,D). The black curve is the average of stochastic realizations with screening 92.5% of patients at the time of hospital admission. Screening 90% of in-hospital patients with exposure to CDI started on day 100. The effectiveness of patient isolation in preventing disease transmission in hospital is 90% (A,C), and 80% (B,D).

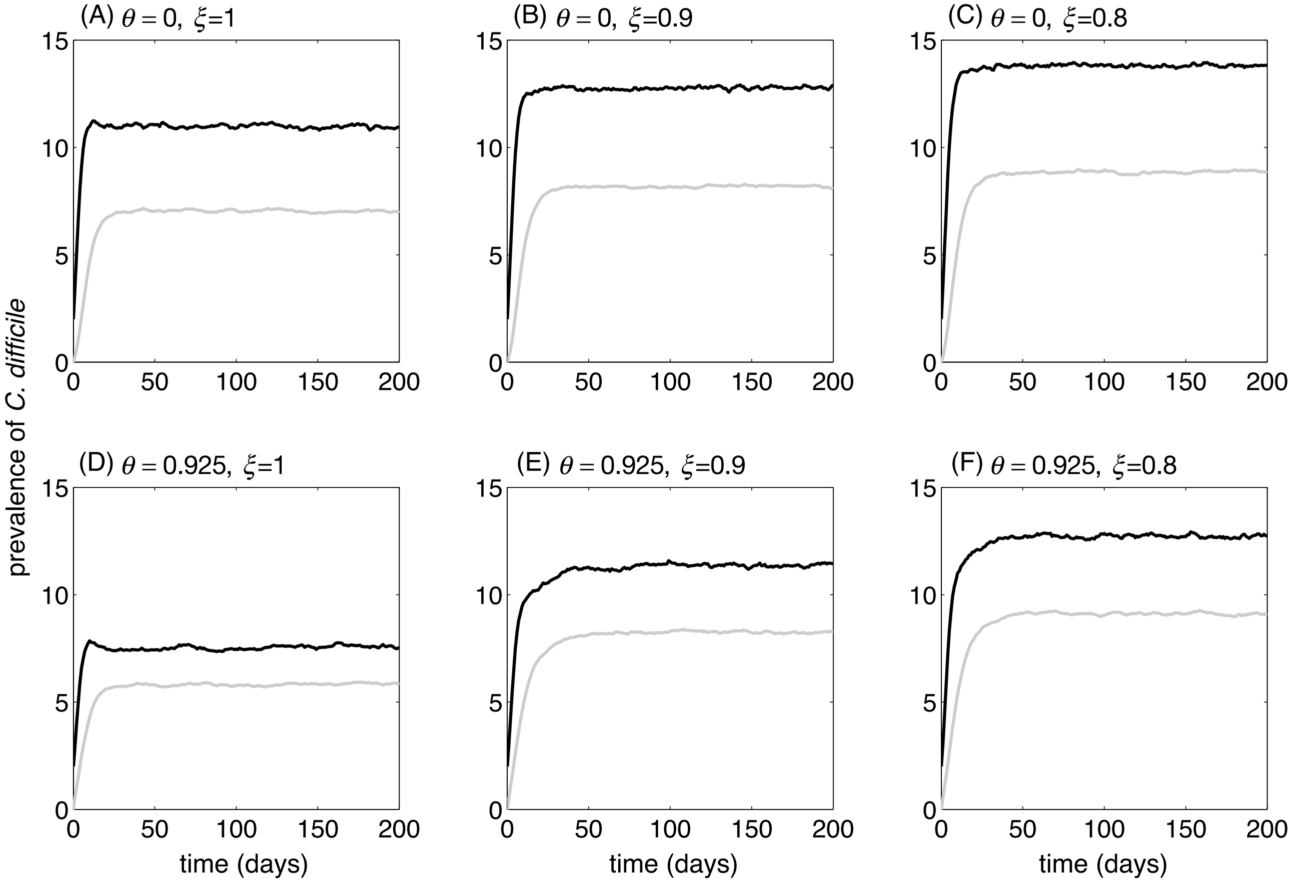

Figure S3: Prevalence of *C. difficile* in the model with rapid laboratory testing, with $R_0 = 2.6$ over 200 days without screening (A-C) and with screening (D-F) 92.5% of patients at the time of hospital admission. Curves represent the prevalence of undiagnosed colonized patients (black), and isolated patients (grey). The total prevalence is the sum of black and grey curves. Effectiveness of isolation for CDI patients was 100% (A,D), 90% (B,E), and 80% (C,F).

Simulation results for $R_0 = 2.6$

Model with rapid laboratory testing

For the mean value of $R_0 = 2.6$, Figure S3 shows the prevalence of *C. difficile* for three scenarios in which the effectiveness of patient isolation in preventing in-ward transmission is 100% (Figure S3A,D), 90% (Figure S3B,E), and 80% (Figure S3C,F). In these simulations, the baseline scenario without screening ($\theta = 0$) was compared with the scenario of 92.5% screening at the time of hospital admission. When the effectiveness of patient isolation is 100%, the prevalence of *C. difficile* reduces from 18 cases without screening to 13.4 cases (on average) with 92.5% screening of patients at the time of hospital admission. This corresponds to approximately 25.8% (95% CI: 25.6% – 25.9%) reduction of prevalence 50 days after the start of screening.

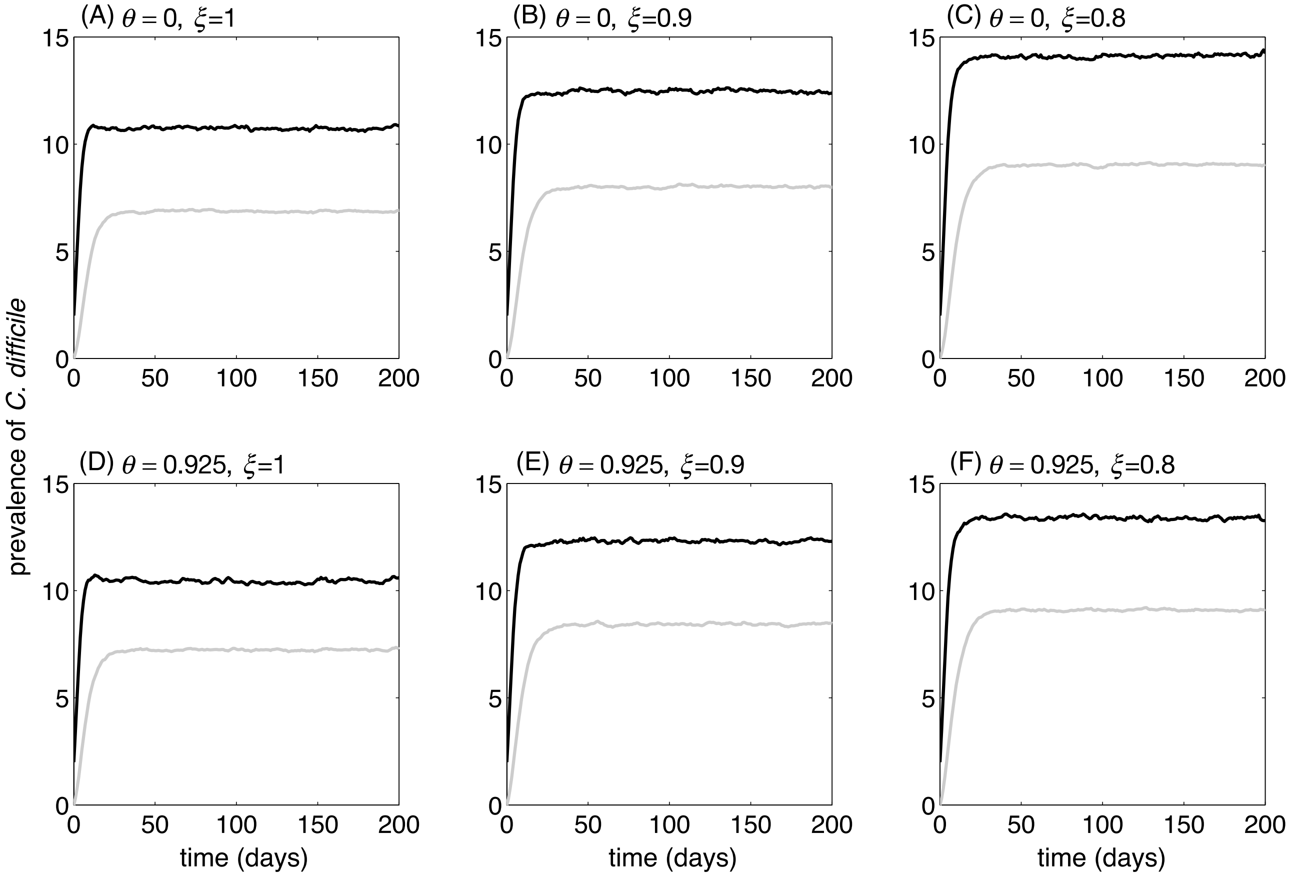

Figure S4: Prevalence of *C. difficile* in the model with a time-delay in laboratory testing, with $R_0 = 2.6$ over 200 days without screening (A-C) and with screening (D-F) 92.5% of patients at the time of hospital admission. Curves represent the prevalence of undiagnosed colonized patients (black), and isolated patients (grey). The total prevalence is the sum of black and grey curves. Effectiveness of isolation for CDI patients was 100% (A,D), 90% (B,E), and 80% (C,F).

For imperfect isolation with less than 100%, the reduction of prevalence is about 6.5% (95% CI: 6.3% – 6.6%) and 3.6% (95% CI: 3.5% – 3.7%) for 90% and 80% effectiveness of patient isolation, respectively.

Model with a time-delay in laboratory testing

Compared with the results for rapid testing, Figure S4 shows a significantly lower effect of patient screening on reducing the prevalence of CDI when an average of 2 days is considered for the time-delay between sample collection and the release of laboratory results. Regardless of the effectiveness of patient isolation, we observed that the reduction of CDI prevalence remains below 1% with screening of patients at the time of hospital admission.

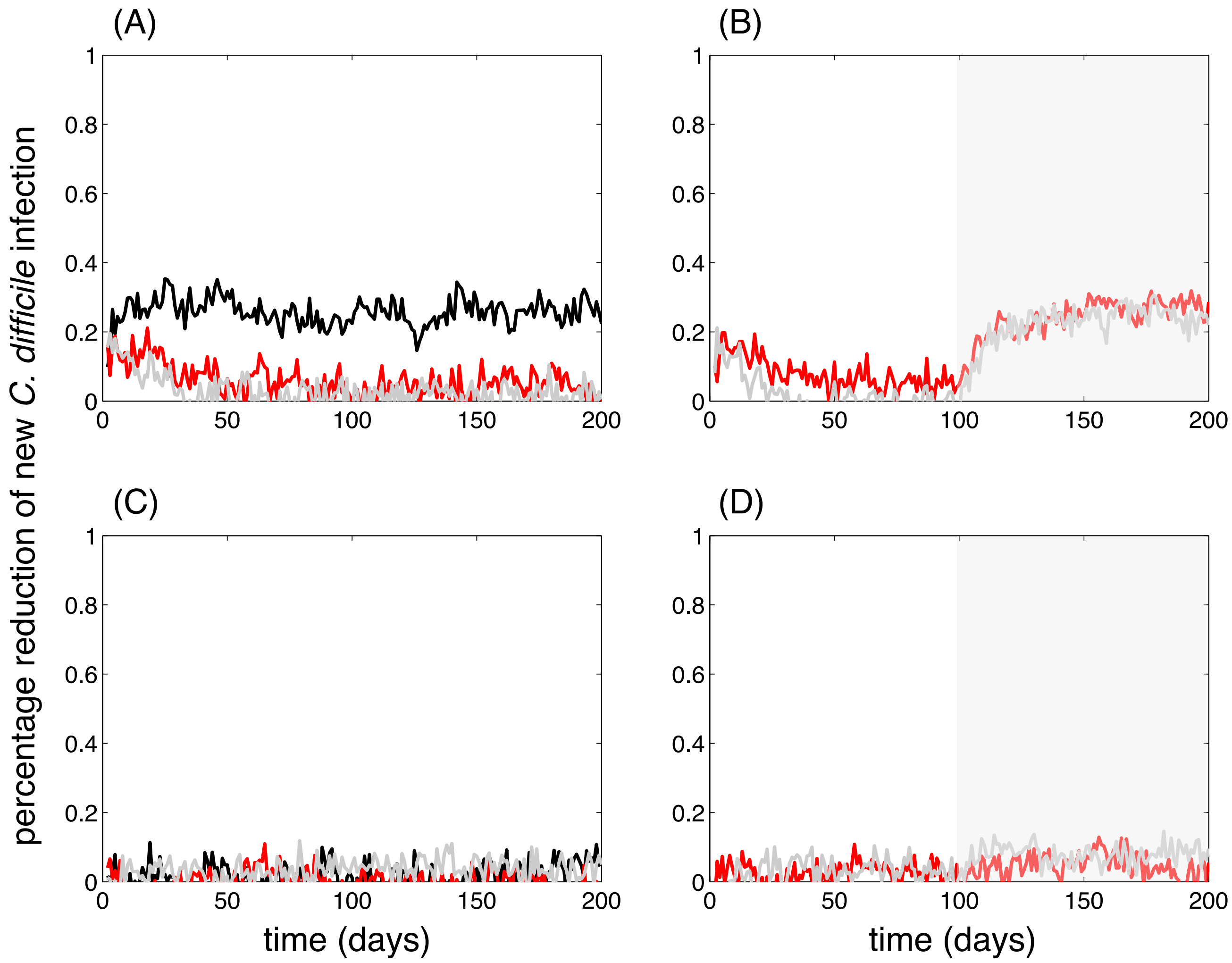

Figure S5: Percentage reduction in the number of new *C. difficile* infection (incidence) with $R_0 = 2.6$ over 200 days for the model with rapid laboratory testing (A,B) and the model with a time-delay in laboratory testing (C,D). In panels (A) and (C), curves represent the reduction achieved with screening 92.5% of patients at the time of hospital admission, where the effectiveness of patient isolation in preventing in-ward transmission is: 100% (black); 90% (red); and 80% (grey). In panels (C) and (D), curves represent the reduction achieved with screening 92.5% of patients at the time of hospital admission, where the effectiveness of patient isolation in preventing in-ward transmission is: 90% (red); and 80% (grey). Screening 90% of in-hospital patients with exposure to CDI started on day 100 (shaded area).

Relative reduction of CDI incidence

Figure S5A (black curve) shows that when the effectiveness of patient isolation is 100% in preventing in-ward transmission in the model with rapid laboratory testing, the daily incidence of *C. difficile* is reduced by over 25.2% on average (95% CI: 24.6% – 25.8%) as a result of 92.5% screening at the time of hospital admission. With lower effectiveness of isolation, this

reduction drops below 4% (Figure S5A, red and grey curves).

For the model with an average of 2 days between sample collection and the release of laboratory results, the relative reduction of incidence is negligible (below 1%) regardless of the effectiveness of patient isolation (Figure S5C).

We then implemented screening of inpatients with exposure of *C. difficile* in addition to screening of patients at the time of admission. For the model with rapid laboratory testing, Figure S5B shows the temporal percentage reduction of the CDI incidence, when screening 90% of in-hospital patients started on day 100. This resulted in an increasing trend in the percentage reduction of *C. difficile* incidence over time (Figure S5B, red and grey curves), reaching levels comparable to those achieved with screening patients only at the time of hospital admission when the effectiveness of patient isolation was assumed to be 100% (Figure S5A, black curve). However, simulating the model with a delay of 2 days in the release of laboratory results indicate marginal benefits, achieving at most 3% increase in the relative reduction of CDI incidence by inpatients screening (Figure S5D).

Sensitivity analysis and PRCC

We carried out a sensitivity analysis using the Latin Hypercube Sampling (LHS) technique and calculated Partial Rank Correlation Coefficients (PRCCs) to investigate the effects of variation in parameter values on the model outcomes (Marino et al., 2008), and specifically on the prevalence of *C. difficile* as the response variable. LHS is a stratified (near-random) sampling technique without replacement. In this method, the random parameter distributions are divided into a number of equal probability intervals, and are then sampled.

The goal of this analysis was to identify key parameters whose uncertainties contribute to prediction imprecision, and to rank these parameters by their relative importance in contributing to this imprecision. To allow for the simultaneous variations of the parameters, samples of size 1000 were generated in which each parameter was treated as a random variable and assigned a probability function. These parameters were sampled using LHS method (near-random sampling) within their respective ranges. To calculate PRCCs, we assumed that there is no correlation between the input parameters (Marino et al., 2008). The parameters with large PRCC values (close to 1 or -1) and their corresponding p-values smaller than the significance level (0.05) have the largest influence on the model outcomes (Taylor, 1990). We examined scatter plots to verify the existence of monotonic relationships between the parameters used in the LHS sampling and the response variable (Figures S6–S9). The PRCC values and their associated p-values are presented in Table S1 and S2 for reproduction numbers of $R_0 = 1.07$ and $R_0 = 2.6$, respectively. The relative influence of model parameters with $R_0 = 1.07$ is summarized in Table 1 of the main text. The relative influence of model parameters with $R_0 = 2.6$ is summarized in Table S3.

References

Pauline van den Driessche and James Watmough. Reproduction numbers and sub-threshold endemic equilibria for compartmental models of disease transmission. *Mathematical biosciences*, 180(1):29–48, 2002.

Simeone Marino, Ian B Hogue, Christian J Ray, and Denise E Kirschner. A methodology for performing global uncertainty and sensitivity analysis in systems biology. *Journal of theoretical biology*, 254(1):178–196, 2008.

Richard Taylor. Interpretation of the correlation coefficient: a basic review. *Journal of diagnostic medical sonography*, 6(1):35–39, 1990.

Table S1: Parameter ranges used in the LHS analysis, partial rank correlation coefficients and their associated p-values with reproduction number of 1.07.

parameter	range	M1[a]		M2[b]	
		PRCC	p–value	PRCC	p–value
f	0.45–0.75	0.171	< 0.001	0.191	< 0.001
κ	0.3–0.7	0.366	< 0.001	0.436	< 0.001
ν	0.3–0.7	−0.876	< 0.001	−0.906	< 0.001
ψ	0.2–0.56	0.399	< 0.001	0.503	< 0.001
α	0.15–0.29	0.036	0.259	0.077	0.016
σ	0–1	−0.363	< 0.001	−0.134	< 0.001
θ	0–1	−0.712	< 0.001	−0.224	< 0.001
ϵ	0.14–0.26	−0.129	< 0.001	−0.002	0.945
τ^+	0.011–0.01	−0.007	0.823	−0.037	0.239
q	0.56–1	−0.467	< 0.001	−0.473	< 0.001
μ_I	0.001–0.01	0.029	0.353	−0.034	0.280
ρ	0.143–0.33	0.138	< 0.001	0.214	< 0.001
γ	0.143–0.33	−0.233	< 0.001	−0.215	< 0.001
ξ	0.8–1	−0.456	< 0.001	−0.631	< 0.001
b_s	0.9–0.99	−0.802	< 0.001	−0.874	< 0.001
η	0.5–0.7	−0.138	< 0.001	−0.108	< 0.001
π	0.33–1	—	—	−0.423	< 0.001

[a] Model with rapid laboratory testing

[b] Model with a time-delay in laboratory testing

Table S2: Parameter ranges used in the LHS analysis, partial rank correlation coefficients and their associated p-values with reproduction number of 2.6.

parameter	range	M1[a]		M2[b]	
		PRCC	p–value	PRCC	p–value
f	0.45–0.75	-0.420	< 0.001	-0.431	< 0.001
κ	0.3–0.7	0.483	< 0.001	0.358	< 0.001
ν	0.3–0.7	-0.910	< 0.001	-0.904	< 0.001
ψ	0.2–0.56	0.857	< 0.001	0.865	< 0.001
α	0.15–0.29	0.119	< 0.001	0.079	< 0.002
σ	0–1	-0.500	< 0.001	-0.037	0.235
θ	0–1	-0.286	< 0.001	-0.015	0.624
ϵ	0.14–0.26	0.073	< 0.020	0.114	< 0.001
τ^+	0.011–0.01	0.188	< 0.001	0.225	< 0.001
q	0.56–1	-0.489	< 0.001	-0.498	< 0.001
μ_I	0.001–0.01	-0.061	0.055	-0.093	< 0.004
ρ	0.143–0.33	-0.273	< 0.001	-0.345	< 0.001
γ	0.143–0.33	0.013	0.672	0.103	< 0.002
ξ	0.8–1	-0.474	< 0.001	-0.665	< 0.001
b_s	0.9–0.99	-0.258	< 0.001	-0.403	< 0.001
η	0.5–0.7	-0.093	< 0.004	-0.053	0.095
π	0.33–1	—	—	-0.356	< 0.001

[a] Model with rapid laboratory testing
[b] Model with a time-delay in laboratory testing

Table S3: Relative influence of the model parameters on the response (i.e., CDI prevalence) based on their PRCC indices and p-values below the significance level in the sensitivity analysis with $R_0 = 2.6$

	relative influence					
**Model**	**rapid laboratory testing**			**time-delay in laboratory testing**		
parameters	strong	moderate	weak	strong	moderate	weak
f		●			●	
κ		●				●
ν	●			●		
ψ	●			●		
σ		●				—
θ			●			●
q		●			●	
ρ			●			●
γ			—			●
ξ		●			●	
b_s			●		●	
η			●			—
π						●

Figures S6-S9 shows the scatter plots of partial residual of parameters used in the sensitivity analysis, corresponding to reproduction numbers $R = 1.07$ and $R_0 = 2.6$, and models with rapid testing and with a time-delay in the release of laboratory results.

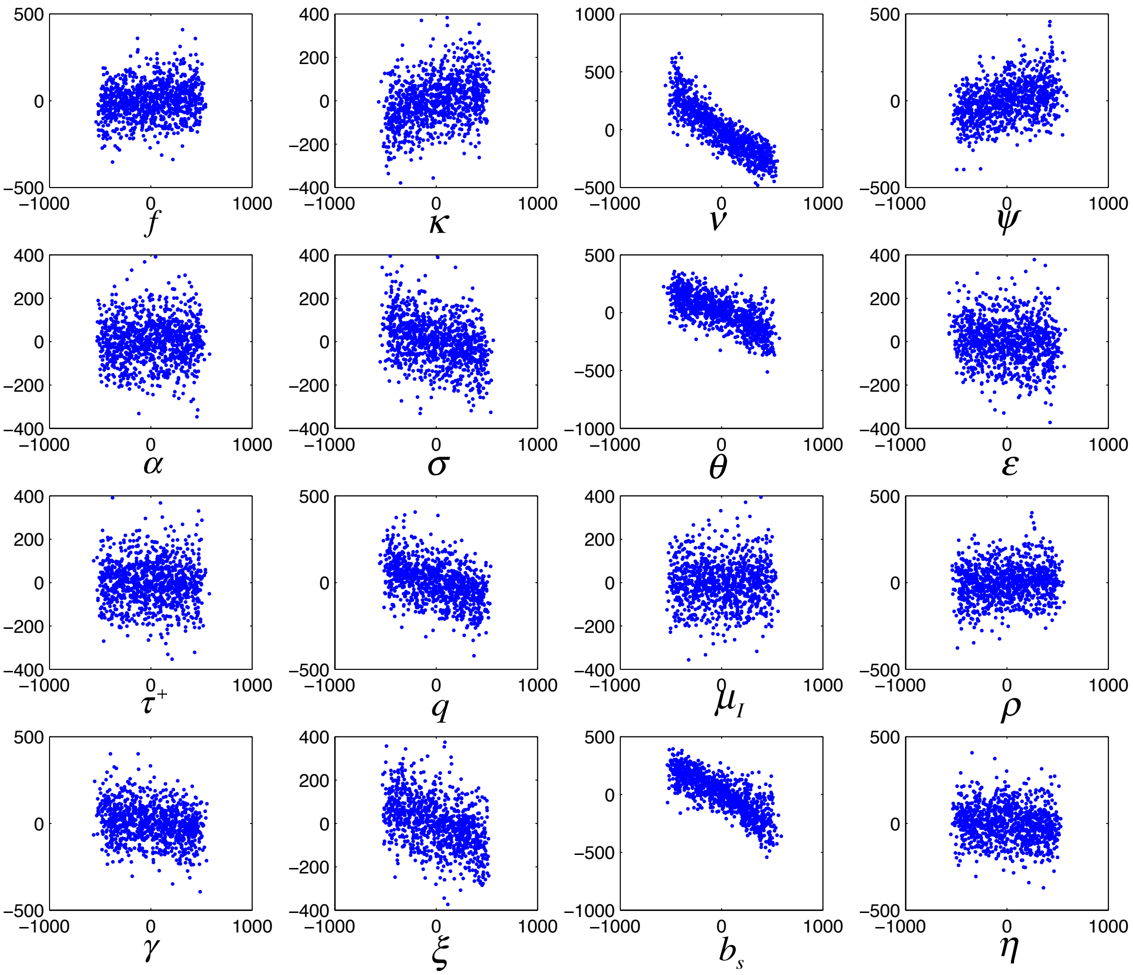

Figure S6: PRCC scatter plots for $R_0 = 1.07$ in the model with rapid laboratory testing. The abscissa represents the residuals of the linear regression between the rank-transformed values of the parameter under investigation versus the rank-transformed values of all the other parameters. The ordinate represents the residuals of the linear regression between the rank-transformed values of the response versus the rank-transformed values of all the parameters under investigation.

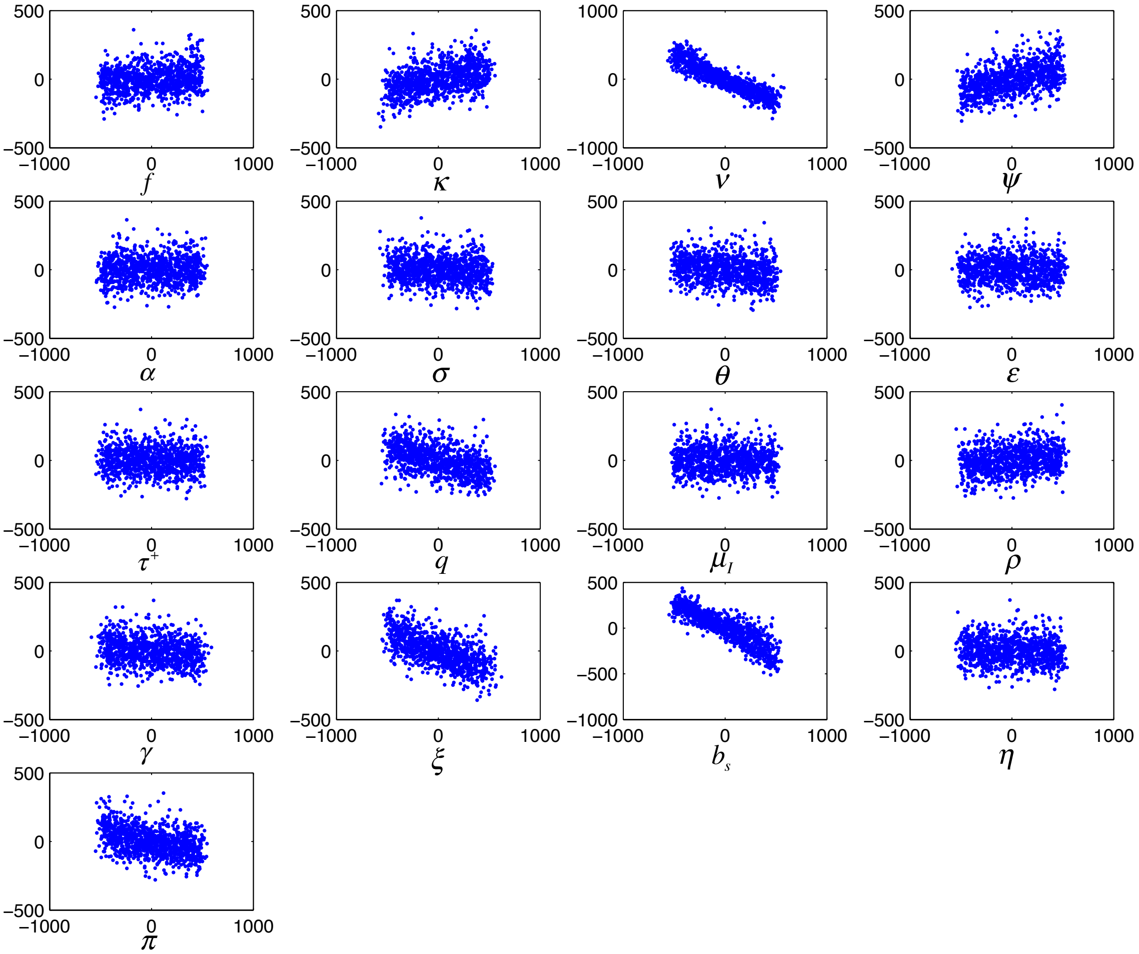

Figure S7: PRCC scatter plots for $R_0 = 1.07$ in the model with a time-delay between sample collection and the release of laboratory results. The abscissa represents the residuals of the linear regression between the rank-transformed values of the parameter under investigation versus the rank-transformed values of all the other parameters. The ordinate represents the residuals of the linear regression between the rank-transformed values of the response versus the rank-transformed values of all the parameters under investigation.

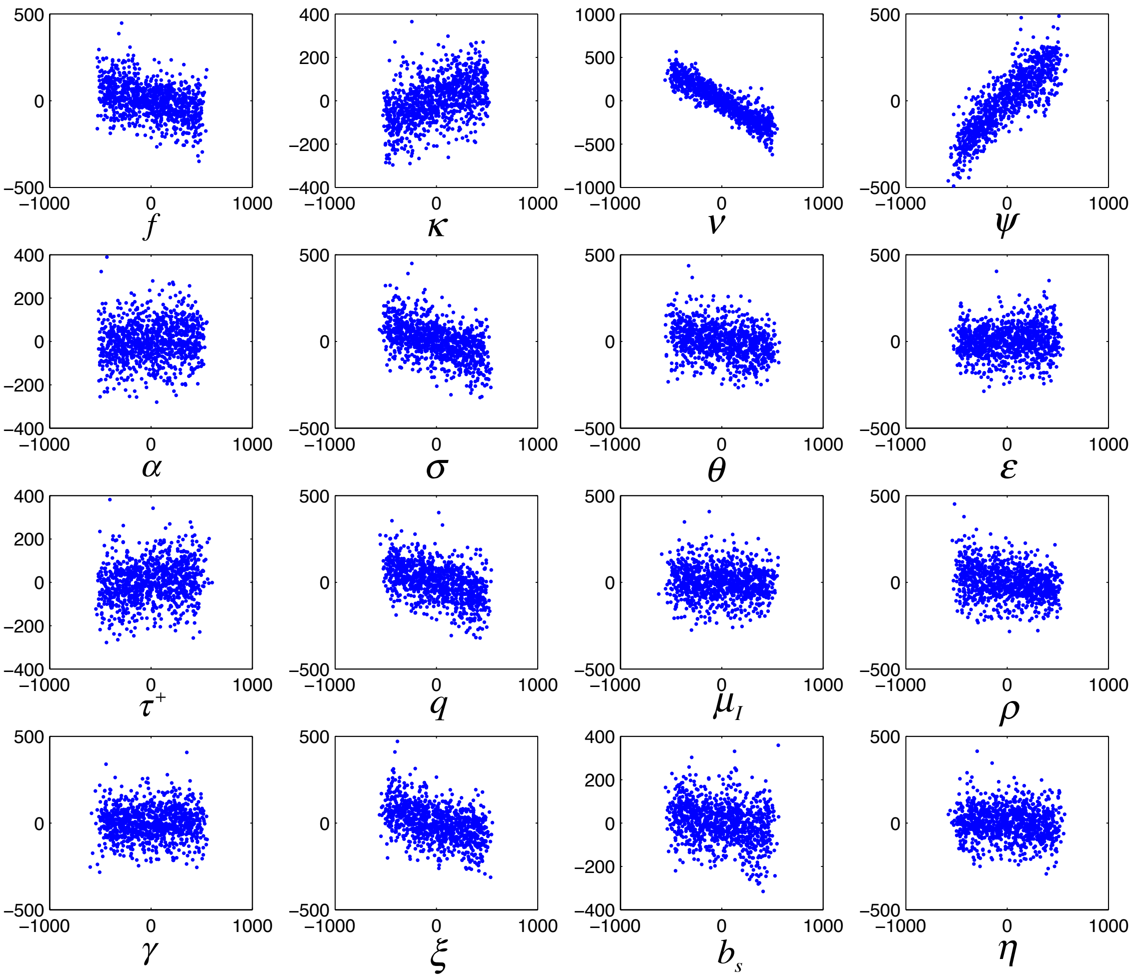

Figure S8: PRCC scatter plots for $R_0 = 2.6$ in the model with rapid laboratory testing. The abscissa represents the residuals of the linear regression between the rank-transformed values of the parameter under investigation versus the rank-transformed values of all the other parameters. The ordinate represents the residuals of the linear regression between the rank-transformed values of the response versus the rank-transformed values of all the parameters under investigation.

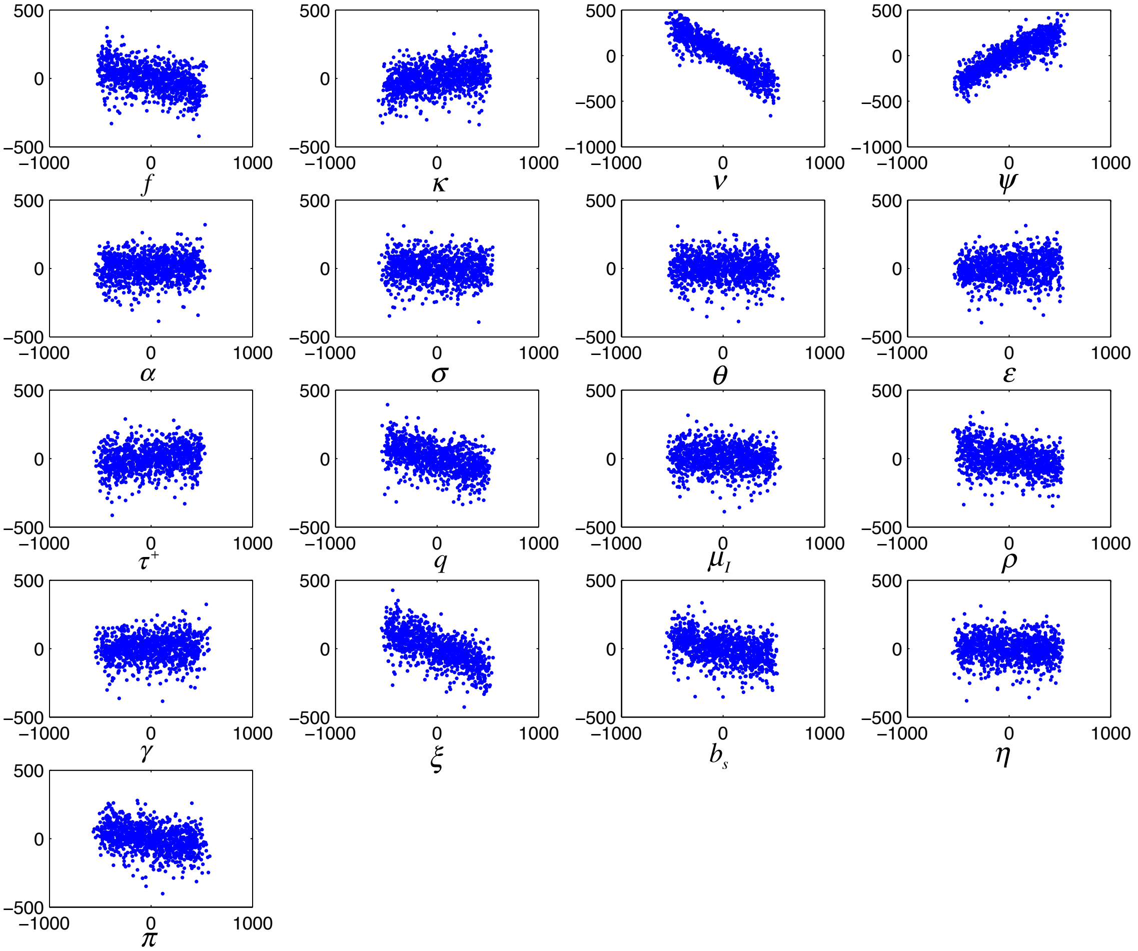

Figure S9: PRCC scatter plots for $R_0 = 2.6$ in the model with a time-delay between sample collection and the release of laboratory results. The abscissa represents the residuals of the linear regression between the rank-transformed values of the parameter under investigation versus the rank-transformed values of all the other parameters. The ordinate represents the residuals of the linear regression between the rank-transformed values of the response versus the rank-transformed values of all the parameters under investigation.
